# Beyond Acute Coronary Syndromes: Troponins as Diagnostic and Prognostic Tools in Heart Failure

**DOI:** 10.3390/biomedicines13102330

**Published:** 2025-09-24

**Authors:** Berenika Jankowiak, Jakub Wilk, Michał Wilk, Aleksandra Orłowska, Piotr Gajewski

**Affiliations:** 1 Institute of Heart Diseases, Wroclaw Medical University, 50-376 Wroclaw, Poland; dr.piotr.gajewski@gmail.com; 2 Student Scientific Organization, Institute of Heart Diseases, Wroclaw Medical University, 50-376 Wroclaw, Poland; w1lkur22@gmail.com (J.W.); michalwilk200218@wp.pl (M.W.); 3 Institute of Heart Diseases, University Clinical Hospital, 50-556 Wrocław, Poland; aleksandra.orlowska@usk.wroc.pl

**Keywords:** heart failure, cardiac troponins, high sensitivity cardiac troponins

## Abstract

Heart failure (HF) is a challenging syndrome, affecting 64 million people worldwide, and is associated with high mortality rates. There are several potential biomarkers that help us diagnose the disease as well as help us assess the prognosis. Cardiac troponins I and T are effective tools for predicting poor outcomes in HF. Cardiac troponins are intracellular proteins responsible for regulating and conducting muscle contractions. They indicate cardiomyocyte injury caused by ischemia, toxins, inflammation, wall stress, and other factors. Some of these injuries may be reversible. The blood level of troponins is influenced by various factors, both physiological—such as sex, age, and weight—and pathological, including microbiological organisms, autoantibody complexes, and conditions like pulmonary embolism, stroke, sepsis, or kidney disease. Numerous studies demonstrate the effectiveness of troponin measurement in prognostication for patients with acute and chronic HF. However, it has been found that many patients with chronic heart failure have undetectable serum levels of cardiac troponins, leading to the adoption of high-sensitivity cardiac troponins (hs-cTn). Expanding this knowledge is crucial for enabling more intensive stratification and early identification of patients with poorer prognoses.

## 1. Introduction

Heart failure (HF) is an intricate clinical syndrome that is characterized by the heart’s inability to receive or eject enough output and/or increased intracardiac pressure due to structural or functional abnormalities. Not only does hemodynamic impairment adversely affect the cardiovascular system in HF but neurohormonal activation also promotes myocardial stress and cell damage, which influence the underlying pathophysiological states in HF. That is why bio-markers such as troponins can be of assistance in the diagnosis, prognostication, and risk stratification of HF. HF is one of the most common causes of hospitalization in people over 65 years of age. It constitutes a significant global issue that continues to grow, especially in developed countries. Despite its prevalence, official guidelines for the treatment of HF have not yet been established. Some European doctors, including general practitioners (GPs), internal medicine doctors and even cardiologists, might not be implementing suggested treatment programs that could improve patients’ clinical state or even prevent them from early death and lower the costs of unnecessary hospitalizations. Therefore, steps have been taken to embrace HF pharmacological therapy. We have pinpointed the methods required to develop and popularize guidelines-dependent treatment. These include creating a national HF register to standardize HF care, educate patients, caregivers and doctors of different specialties on the newest strategies and expand access to specialized units and novel treatments [1,2]. HF is associated with high mortality and morbidity, as well as substantial costs. Therefore, it is crucial to improve diagnostics and methods for prognostication in these patients.

Several potential biomarkers, such as bicarbonates, IL-6, IL-17 and VEGF-C, assist in diagnosing the disease and assessing its prognosis, in addition to grading or phenotyping the disease [3,4,5,6,7,8,9,10].

Elevated levels of troponins in blood have been detected in nearly 60% of patients admitted to hospital with acute heart failure (AHF). Patients with significant increases in hs-TnI were more likely to suffer a CV-related death [10]. Cardiac troponins I (cTnI) and T (cTnT) are used as a primary diagnostic tool for acute coronary syndromes (ACSs) [11]; however, their elevated levels are not exclusive to myocardial ischemia. Measuring these biomarkers has proven to be an effective tool in risk assessment for patients with heart failure, first described in 1997 [12], as their elevation specifically correlates with cardiomyocyte injury. It is important to emphasize that troponins are associated not only with cardiomyocyte death or pathology but also with natural aging and intensive training, and this condition may be reversible [13]. Their high levels are linked to an increased risk of poor outcomes among HF patients, even in the absence of ACS, and are observed in both AHF and chronic heart failure (CHF), thereby retaining prognostic value when combined with natriuretic peptides and other biomarkers. Nevertheless, the level of troponins in HF is lower than in patients with ACS. Many patients with a lower severity of HF have undetectable levels of cardiac troponin, which led to the introduction of highly sensitive cardiac troponins (hsTnT) that detect concentrations more than ten times lower than the threshold of traditional measurements [14].

Troponins are used in prognostic endpoints in heart failure with a preserved ejection fraction (HFpEF) and AHF with a predominantly reduced ejection fraction [15,16,17,18,19,20,21].

## 2. Methodology

In this review, we searched the literature in order to identify studies that utilized troponins in the context of heart failure. The first and major database accessed was Wiley Online Library, supplemented by searches in PubMed and other relevant databases where appropriate. The literature search was conducted using relevant MeSH terms, including ‘Troponin I, Cardiac’, ‘Troponin T, Cardiac’, ‘Heart Failure’, ‘Biomarkers’, and ‘Cardiomyocyte Injury’, to identify studies evaluating the clinical utility of circulating cardiac troponins in heart failure. The search included articles published in the database up to the year 2025 and placed no restrictions on study design in order to cover articles addressing the diagnostic, prognostic, and risk stratification aspects of troponins as thoroughly as possible.

Inclusion criteria were peer-reviewed articles, reviews, clinical trials, and observational studies discussing troponins in heart failure. Trials/reports that only focused on acute coronary syndromes without discussion of heart failure were excluded. The selection criteria for the referenced articles were solely based on the relevance to the topic, novelty, and representation of data for clinical utility of troponins for diagnosis, prognosis, and risk stratification in heart failure.

## 3. Physiology and Pathophysiology of cTnI

Cardiac troponin I (cTnI) is a tissue-specific protein, encoded by the TNNI3 gene and expressed in three paralogs of which exclusively one isoform is present in cardiac muscle, where it forms a troponin complex responsible for regulating and conducting muscle contractions [22]. This variant is a 209 amino acid-long polypeptide containing two phosphorylation sites, consisting of two serine residues in position 23 and 24 [23,24]. The mentioned sites are phosphorylated by protein kinase A, as a response to beta-adrenergic signal transduction, enhancing inotropic response. Phosphorylation of cTnI allows a structural shift, which is the fundamental feature of its function [25]. It is well established that troponin function is calcium-dependent; accordingly, at a low cytosolic Ca^2+^ concentration, the actomyosin complex forming a myofilament is inhibited by the resting sterical form of cTnI, which refers to its stable three-dimensional conformation [22,24,25]. When Ca^2+^ ions bind to cardiac troponin C (cTnC), it promotes structural change by exposing the hydrophobic domain towards cTnI. This interaction between cTnC and cTnI launches a hinge-like movement of the troponin complex, allowing retraction of tropomyosin, which results in exposure of the myosin-binding site located on the actin surface [22,24]. Accordingly, proper cTnI function is essential in sustaining basic cardiomyocyte function and the ability to adapt to applied stress factors.

Its presence in the bloodstream is an indicator of cardiomyocyte injury which can be induced by many processes such as ischemia, wall extension, toxins, inflammation, etc. We can divide them into irreversible and reversible. The first category occurs when a cell is destroyed during necrosis or apoptosis [26]. Studies on porcine heart found cTnI release in non-necrotic injured tissue [27]. Reversible cardiomyocyte injury is a more complicated matter. The release of cTn is not always connected to cell death. This process may be defined as a troponin leakage. However, it has been suggested that apoptosis may be primarily responsible for the release of troponins into the blood, even if it is not the only mechanism for their increase [28]. Stressors and their cellular repercussions, if limited/quickly reversed, can cause a small degradation of cTnI, which is evacuated from the cell. This process is available due to the increase in cell membrane permeability, and the formation of blebs (cytoplasmatic vesicles) or microparticles [29]. Another way in which cTnI is released is after intensive physical exercise such as long-distance running. Athlete’s troponin levels have been found to elevate just after intense physical exertion with a peak after 2–4 h and then go back to the normal range 24 h post-exercise [30]; however, in patients with HFrEF, structured exercise intervention was associated with a significant reduction in hs-cTnI [31].

Another aspect is cTn clearance. Released cardiac troponins are catabolized in the liver, pancreas, or kidneys. The clearance rate of troponins in ST-elevated myocardial infarction (STEMI) measured in a recently published article ranged from 40.3 to 77.0 mL/min [32]. If any impairment in function such as renal or liver failure occurs, it can lead to a higher concentration of cardiac troponins in serum.

## 4. Parameters Influencing Changes in cTnI Concentration

Cardiac troponin concentration is a very individual matter, which complicates establishing universal cutoffs. Lately, there has been discussion about creating more specific and tailored individual-patient parameter cutoffs of the 99th percentile. The difference in cTnI concentration between men and women is well recognized. The mass of men’s heart muscle is on average bigger; therefore, more cardiac troponin is synthesized and leaked or released during injury. For example, research on healthy cohorts provided relevant differences between the 99th percentile in males vs. females (10.49 pg/mL and 4.98 pg/mL, respectively) [33]. Another study on a much bigger group demonstrates a lower median cTnI concentration in women than men [34]. High levels of cardiac troponin I in women were correlated with higher cardiovascular disease coincidence than relative concentration in men [34]. Moreover, research on a Japanese population also proved that the hs-cTnI 99th percentile differs between the two genders with the male cutoff being higher than women (30.6 pg/mL and 17.7 pg/mL, respectively) [35]. Another work found sex to be a strong determinant influencing cardiac troponins levels in patients aged 25–41 (15.79 and 5.11 pg/mL for men and women) [36]. They also found age to be a strong determinant of higher cs-cTnI. On the other hand, in the elderly population, sex-specific cutoffs did not differ in the incidence of new cardiovascular events [37].

Another factor influencing cTnI concentration is body weight. Overweight and obesity are strongly correlated with elevation of cardiac troponins due to higher susceptibility to subclinical myocardial injury. The correlation between obesity and cTnI concentration was also found among patients undergoing bariatric surgery [38]. Patients were found to have lower cardiac troponin levels after surgery. There seems to be some evidence linking race and the concentration of cardiac troponins but it needs to be studied more extensively.

## 5. Comorbidities Associated with cTnI Changes

Although cTnI is considered the gold standard of monitoring myocyte necrosis, it is important to think about the multitude of mechanisms in which this isoform of troponin can be elevated. Numerous non-cardiac conditions can lead to a surge in cTnI concentration, where various pathological backgrounds can be observed. In many patients, non-cardiac or multiple cardiac conditions can coexist with HF, influencing the cTnI concentration and hindering the interpretation of laboratory assessment [39,40,41]. We can identify some diagnostic patterns that could support our view on interpreting this parameter correctly.

Firstly, the direct cytotoxic effect of infectious factors, such as bacteria, viruses, fungi, or parasites, is a frequent cause of troponin elevation [39,40]. It is well documented that conditions like pericarditis, myocarditis, and endocarditis are associated with cTnI elevation; even though pericardium and endocardium do not contain troponin molecules inside their cells, cTnI is still heightened due to the involvement of myocardium in the inflammatory process [39,42]. A similar effect can be caused by autoantibody complexes as in systematic lupus erythematosus [43], antibody-mediated targeting of postsynaptic receptors in myasthenia gravis [44], or another unknown autoimmune mechanism as in systematic scleroderma (SSc) [45]. Regarding autoimmune diseases that affect heart muscle, it has been shown that immunoassays may generate analytical errors; thus, they need to be evaluated cautiously and may require support from other parameters [46].

Pulmonary embolism (PE) may also lead to cTnI elevation [39,40,47]; it allows us to determine the patients that are presumably going to need mechanical ventilation or inotropic support. Also, indirectly, cTnI allows us to distinguish patients at particular risk of cardiogenic shock, increased risk of death and adverse outcomes [40]. Decreased GFR in chronic kidney disease (CKD) contributes to troponin elevation through mechanisms like uremic skeletal myopathy and impaired protein metabolism. Additionally, cardiorenal syndrome leads to volume overload and cardiac hypertrophy, potentially causing myocardial ischemia and increased cTnI release [40,47,48,49]. Another condition associated with increased cTnI release is sepsis [40,47,50,51,52,53]. Interestingly, central nervous system (CNS) diseases, like stroke or subarachnoid hemorrhage, are considered a significant risk factor for troponin elevation [40,41,54,55].

## 6. Cardiac Troponin I in Acute Heart Failure

AHF is a heterogeneous clinical state associated with rapid onset or exacerbation of characteristics for HF symptoms [56]. In relation to the systematic nature of this condition, and correlative multiorgan dysfunction, it requires urgent hospitalization, diagnostics, and treatment for the purpose of improving patients’ outcomes [56]. In-hospital mortality reaches 4–10%, and one-year mortality can be as high as 30%, which shows the seriousness of this clinical situation [6].

Around one-third of AHF cases are de novo, while most result from worsening chronic heart failure, often due to ischemic, inflammatory, or cardiotoxic factors. In cases without an identifiable cause, thorough medical evaluation, including troponin assay, is crucial, as recent studies highlight the diagnostic value of high-sensitivity troponin I [39,40,41,56,57,58,59,60,61,62,63,64,65,66]. Routinely measured cTnI in AHF patients is often elevated [59,64,65,66]. The rapid development of troponin assay and the introduction of high-sensitivity cardiac troponin tests have significantly changed our approach to how troponin should be perceived in clinical interpretation. Even though troponin testing is not used for the diagnosis of AHF, serum troponin levels can serve as a predictive factor for various outcomes. Troponins appear to be highly effective in the prognosis of AHF, with the greatest predictive value attributed to hs-TnI and hs-TnT [64]. Since hs-cTnI is detectable in about 50% of asymptomatic individuals, it should be seen as a prognostic variable rather than a strict cut-off marker as it is useful even though it is under 99th percentile upper reference level (URL) [59].

In the study by Xue et al. [66], 144 patients underwent laboratory testing to assess hs-TnI levels in the context of AHF. Over 99% of these patients had hs-TnI levels above the 99th percentile of the reference population. An hs-TnI level exceeding 23 ng/L was associated with an increased risk of hospitalization and death [66]. Moreover, recent studies have also shown that patients with any detectable concentration of hs-cTnI are as well at higher risk of death [41,59,61,63]. The ADHERE study analyzed 67,924 decompensated HF patients with serum creatinine levels below 2.0 mg/dL. Troponin I levels were measured in 61,379 patients, and troponin T in 7880 patients; both types were measured in 1335 patients [16]. Troponin levels exceeding 0.1 µg/L were considered positive, which was observed in 6.2% of the cohort. Among these troponin-positive patients, 8% died in the hospital compared to 2.7% of troponin-negative patients (*p* < 0.001) [65]. The adjusted odds ratio for mortality among troponin-positive patients was 2.55 (95% CI, 2.24–2.89, *p* < 0.001). Additionally, ischemic heart failure was observed in 53% and 52% of troponin-positive and troponin-negative patients, respectively, and was not associated with higher mortality, indicating that it cannot be considered a discriminator of troponin status. Troponin positivity in AHF patients is also associated with longer hospital stays and higher resource use in the intensive care units [16]. The strongest correlation between mortality and troponin-I positivity is observed over the first 30 days after admission [16,60,61]. In troponin-negative patients, the 30-day mortality is estimated as 3%, where for troponin-positive patients, below the 99th URL, it is 9%. However, the worst outcomes are observed in the troponin-positive subset above the 99th URL where the 30-day mortality is around 20% [60]. In the case of long-term mortality defined as an endpoint at 180 days after hospital admission in patients with AHF and cTnI positivity, the correlation is maintained, but it is much weaker [61]. A detectable hs-cTnI concentration was also identified as a risk factor for abnormal ECG patterns in the AHF population [67]. In this subset, appearance of complex premature ventricular contractions was increased, which can also be relevant clinically in terms of future complications, but it needs further investigation [67]. This evidence advocates for the great prognostic value provided by cTnI assays in terms of stratifying risk and potentially modifying therapeutic strategies for more aggressive ones in order to react against possible exacerbation of AHF in a particularly susceptible subset of patients early on [59,60,61,62,63,64,65,66]. In a study by Alhejily et al. [15], high-sensitivity cardiac troponin I was measured in 95 patients with suspected acute pulmonary edema due to AHF and elevated pro BNP levels exceeding 1000 pg/mL. The authors used hs-cTnI to predict outcomes such as heart failure-related mortality and poor prognoses. Despite the small cohort size, the results highlighted the advantages of hs-cTnI compared to pro BNP, showing it to be more specific and sensitive in predicting the risk of death and adverse outcomes [15]. This study demonstrated statistical significance (*p* = 0.001). Despite comparable mean levels of pro BNP and ejection fraction, hs-cTnI showed odds ratios of 8.5 and 4.3 for predicting mortality and poor outcomes, respectively [15]. Routinely measured cardiac troponin I is a superior marker of cardiomyocyte damage in AHF and should be a key factor in therapeutic decisions rather than just a supplementary diagnostic tool [15,60]. It is important to emphasize that the prognostic value of the cTnI assay in AHF is strongest at the beginning of hospitalization and becomes weaker over time; hence, it has to be evaluated as fast as possible [64,66].

It is also important to remember that elevated hsTnI during AHF episodes can be associated with acute coronary syndrome (ACS). Hence, Serenelli et al. proved that change in troponin levels defined as the baseline measured at hour 0 and second measurement at the 3rd hour after admission may be an important factor in differentiation between those two conditions. Those with severe HF usually had higher baseline hsTnI levels, which could mask its change in ACS [68].

## 7. Cardiac Troponin I in Chronic Heart Failure

CHF is a syndrome characterized by symptoms resulting from impaired cardiac function. CHF patients are those in whom heart failure has developed gradually or existed previously. This condition requires long-term treatment, regular medical follow-ups, and both laboratory and imaging studies to monitor disease progression and respond promptly if the condition transitions to AHF [6].

Not all CHF patients will have detectable levels of cardiac troponins in their blood; however, their presence predicts worse outcomes for the patient [14,59,69].

Wei et al. presented in their study a connection between the mechanical stretch of cardiomyocytes, which is common in HF, leading to their injury, and how this accounts for elevated troponin levels without ischemia or necrosis [27].

The EMPEROR-Preserved study, conducted on 5988 patients, developed a prognostic model for CHF patients with a preserved ejection fraction (HFpEF), focusing on biomarkers such as NT-proBNP and high-sensitivity cardiac troponin T (hs-cTnT). The study evaluated outcomes including primary outcomes of HF hospitalization or cardiovascular death, and secondary outcomes of all-cause mortality, HF hospitalization, and cardiovascular death. This model achieved c-statistics of 0.711 (95% CI 0.672–0.749) for cardiovascular death or HF hospitalization, 0.719 (95% CI 0.665–0.772) for all-cause death, and 0.718 (95% CI 0.652–0.784) for cardiovascular death. These results indicate that the model effectively discriminates patients at higher versus lower risk of cardiovascular death or HF hospitalization, rather than actually reducing the risk [70].

The ARISTOTLE substudy [71] highlighted the superiority of troponin I in predicting poor outcomes in patients with HF, atrial fibrillation (AF), and vascular diseases. Troponin T levels were more strongly associated with factors such as diabetes, age, and male sex. This study involved 14,806 patients with AF.

A study conducted by Suzuki et al., which involved 155 patients with HF, has proven that those with HFpEF (EF > 50%) and elevated hsTnT were more prone to experience adverse events [72].

Research by McDowell et al. [73], involving 6263 participants, aimed to create a model incorporating clinical and laboratory variables to predict morbidity and mortality in HFpEF patients. The 1-year C-statistics for cardiovascular death and all-cause mortality were 0.73 (95% CI, 0.71–0.75) and 0.71 (95% CI, 0.68–0.74), respectively. At 2 years, they were 0.71 (95% CI, 0.68–0.74) and 0.68 (95% CI, 0.66–0.70). HF hospitalization (HFH) risk varied across cohorts, with the DELIVER trial achieving a C-statistic of 0.72 (95% CI, 0.70–0.74) and the PARAGON-HF trial reporting 0.66 (95% CI, 0.64–0.67).

Similarly, the GALACTIC-HF study examined HF patients with reduced ejection fraction (HFrEF), showing that patients with poorer outcomes had higher troponin I levels. The hazard ratio for the primary endpoint (first HF event or cardiovascular death) was 1.30 (95% CI 1.28–1.33; *p* < 0.001 per doubling of baseline cTnI) [74].

Troponins have been studied not only as predictors of clinical outcomes but also, along with NT-proBNP, as screening tools for subclinical CHF [75]. Averina et al. [76] explored this concept using sex- and age-specific upper reference limits (99th percentiles for hs-troponin T and 97.5th percentiles for NT-proBNP) in a cohort of generally healthy patients with normal echocardiographic findings and no conditions that could distort the results. Exclusion criteria included myocardial infarction, angina pectoris, atrial fibrillation, HF, stroke, diabetes mellitus, hypertension, obesity, moderate/severe anemia, and kidney disease (GFR ≤ 60 mL/min/1.73 m^2^). However, this approach proved insufficient as a reliable screening method.

Among patients with HFpEF, both hs-TnT and hs-TnI are elevated, but the predictive value is stronger for hs-TnT. The prognostic significance of hs-cTn is associated with poorer outcomes in men compared to women, with hs-TnI being a better marker for the male sex. Additionaly, mild renal function decline in HFpEF patients is associated with significant cTnI serum concentration elevation [77]. The aforementioned findings provide valuable insights into tailoring treatment intensity for HF patients. Those with worse outcomes should be treated more aggressively to mitigate risks and improve prognoses.

## 8. Cardiac Troponin I in Heart Failure: Insights into Amyloidosis and Hypertrophic Cardiomyopathy

### 8.1. Hypertrophic Cardiomyopathy (HCM)

HCM is a condition caused by primary mutations of various genes coding structural proteins of sarcomere, for instance, troponins [78,79,80]. A subtype of this mutation is associated with abnormalities within the troponin I coding gene called TNNI3 [78,79]. Despite the fact that this mutation accounts for around 3% of all HCM cases, its role seems to be pivotal to better understanding the complex dependencies between HCM and cTnI [81]. Although this connection is poorly examined, different information regarding the above-mentioned subject has emerged. Firstly, a study conducted by Abood et al. confirmed that elevated concentrations of hs-cTnI are observed in 17% of patients suffering from obstructive HCM, proving that troponin I might have a diagnostic value in detecting this condition [82]. Another study by Zhang et al. expanded related findings by confirming that patients with elevated cTnI concentrations had worse outcomes and prognoses, especially if cTnI was combined with CK-MB elevation simultaneously [83]. The authors emphasize that guided and routinely performed diagnostics of cardiac troponin I represent a suitable tool in the implementation of primary prevention in the form of an implantable cardioverter defibrillator (ICD) [83]. As a result, an improved HCM Risk-SCD model was proposed that proved to be a more successful tool in SCD (sudden cardiac death) prevention among HCM patients [83]. The aforementioned findings are further supported with a subsequent study by Osmanska et al. stating that cTnI elevation in HCM is associated with higher mortality [84]. This is explained as a result of more advanced LV fibrosis, higher LV mass, and left ventricle outflow tract (LVOT) gradient in the HCM subset [84]. The pathogenesis of this condition is probably caused by disturbed myofilament kinetics, which in turn results from calcium mishandling and the impaired ability to relax [80,81]. Therefore, cTnI seems to be an important tool in both the diagnosis and stratification of HCM; however, this subject requires further study.

### 8.2. Cardiac Amyloidosis

A significant challenge in contemporary cardiology is amyloidosis, a condition characterized by the deposition of misfolded proteins in various tissues, including the myocardium. Of clinical importance is the role of amyloidosis as an underlying etiology of heart failure. The two most common types of cardiac amyloidosis are immunoglobulin light chain (AL) amyloidosis and transthyretin-related (ATTR) amyloidosis. High-sensitivity cardiac troponins (hs-cTnT) have proven effective as prognostic biomarkers in amyloidosis, as elevated levels are frequently observed in affected patients [85,86,87,88,89,90,91].

High-sensitivity troponins, next to natriuretic peptides, can be very useful as predictors in treatment response, disease staging, and survival of AL amyloidosis. For the sake of simplicity, the higher the troponins, the worse the prognosis [92,93]. Troponins have also been used in ATTR-CA. Asymptomatic patients presenting with elevated levels of these biomarkers, in the absence of an alternative identifiable cause, should undergo diagnostic evaluation for transthyretin cardiac amyloidosis (ATTR-CA) to facilitate early diagnosis and timely intervention [94,95,96]. Be that as it may, their role in prognosis in this condition has not been comprehensively evaluated yet, although we have reports about their predicting role in the final diagnosis [94,95,96].

## 9. Conclusions

There are many predictive factors in heart failure, such as NT-proBNP, and echo-measured ventricular and/or atrial dimensions, helping physicians to foresee the outcome. Cardiac troponins and their predictive factors are mostly connected with myocardial necrosis in ACS. Recently, many authors and clinical trials have been focused on the usage of cardiac troponins in heart failure as demonstrated in Table 1. Nevertheless, lately, many authors and clinical trials have been focused on the usage of cardiac troponins in heart failure. Many of them proved that cTnI and cTnT can be used to predict the risk and poor outcome in both AHF and CHF. Moreover, other clinical states such as PE, AF, CKD, and CNS are also connected with cardiac troponin elevation. This study and trials are proof that we are in need of a new stratifying tool that could connect cTnI and its prognostic value in patients with AHF and CHF.

## Figures and Tables

**Table 1:** Summary of major insights regarding troponins in heart failure.

Author, Year	Population Studied	Biomarker(s) Studied	Main Findings	Comment
Horwich et al., 2003 [63]	Advanced CHF	cTnI	Higher cTnI associated with worse hemodynamics and increased mortality	Troponin I useful for stratifying advanced HF patients
Latini et al., 2007 [14]	Stable CHF	hs-cTnT	Very low hs-cTnT levels still have prognostic value	Adds value to NP in risk assessment
Peacock et al., 2008 [16]	AHF in emergency department	cTnT, NT-proBNP	cTnT independently predicts 60-day mortality	NT-proBNP better for diagnosis, troponin for prognosis
Xue et al., 2011 [66]	Decompensated HF	hs-cTnI, NT-proBNP	Serial hs-cTnI changes predict worse outcomes	Serial troponin better than single measurement
Felker et al., 2012 [61] (ASCEND-HF)	Decompensated HF	cTnI, BNP	cTnI predicts death and rehospitalization	Troponin complements NP in prognostic models
Arenja et al., 2012 [60]	AHF	hs-cTn, BNP	Troponin sensitive for mortality and rehospitalization risk	Identifies high-risk patients
Pascual-Figal et al., 2012 [17]	AHF	hs-cTnT, NT-proBNP, sST2	Elevated hs-cTnT → higher risk of death and rehospitalization	Adds prognostic value beyond NP and sST2
Suzuki et al., 2019 [72]	HFpEF	hs-cTnT, NT-proBNP	hs-cTnT predicts cardiovascular events	Complementary to NT-proBNP
Yan et al., 2020 [65]	Patients at risk of HF	hs-cTnI	hs-cTnI predicts development of HF	Useful in general population, earlier than NP
Meijers et al., 2021 [20]	All HF patients	Troponins, NP, sST2, GDF-15	Troponin complements other biomarkers	Prognostic value beyond NP
Pocock et al., 2022 [70] (EMPEROR-Preserved)	HFpEF	hs-cTnT, NT-proBNP, GDF-15	Troponin included in prognostic model	Improves model calibration and c-index
Averina et al., 2022 [76]	General population	hs-cTnT, NT-proBNP	hs-cTnT + NT-proBNP better for detecting subclinical HF	Troponin complements NP screening
Castiglione et al., 2022 [64]	All HF patients	cTn, BNP, sST2, galectin-3	Troponin is a prognostic biomarker in HF	Emphasized role in risk stratification
Riveland et al., 2024 [31]	HFrEF + exercise	hs-cTnI	Exercise affects hs-cTnI levels	Monitor troponin during rehabilitation programs
Felker et al., 2024 [74] (GALACTIC-HF)	HFrEF with omecamtiv mecarbil	hs-cTn	Baseline troponin predicts response to therapy	Troponin used to assess treatment effects

List of abbreviations: CHF—chronic heart failure, cTnI—cardiac troponin I, hs-cTnT—high-sensitivity cardiac troponin T, AHF—acute heart failure, NT-proBNP—*N*-terminal pro–B-type natriuretic peptide, hs-cTnI—high-sensitivity cardiac troponin I, BNP—B-type natriuretic peptide, hs-cTn—high-sensitivity cardiac troponin, sST2—soluble suppression of tumorigenicity-2, HFpEF—heart failure with preserved ejection fraction, GDF-15—growth differentiation factor-15, HFrEF—heart failure with reduced ejection fraction.
